# Association of climatic factors with COVID-19 in Pakistan

**DOI:** 10.3934/publichealth.2020066

**Published:** 2020-11-11

**Authors:** Yasir Rehman, Nadia Rehman

**Affiliations:** Canadian Academy of Osteopathy, 66 Ottawa Street North, Canada

**Keywords:** COVID-19, climatic effects, humidity, lock down, temperature, transmission

## Abstract

**Introduction:**

Environmental factors such as wind, temperature, humidity, and sun exposure are known to affect influenza and viruses such as severe acute respiratory syndrome (SARS) and Middle East Respiratory Syndrome (MERS) transmissions. COVID-19 is a new pandemic with very little information available about its transmission and association with environmental factors. The goal of this paper is to explore the association of environmental factors on daily incidence rate, mortality rate, and recoveries of COVID-19.

**Methods:**

The environmental data for humidity, temperature, wind, and sun exposure were recorded from metrological websites and COVID-19 data such as the daily incidence rate, death rate, and daily recovery were extracted from the government's official website available to the general public. The analysis for each outcome was adjusted for factors such as lock down status, nationwide events, and the number of daily tests performed. Analysis was completed with negative binominal regression log link using generalised linear modelling.

**Results:**

Daily temperature, sun exposure, wind, and humidity were not significantly associated with daily incidence rate. Temperature and nationwide social gatherings, although non-significant, showed trends towards a higher chance of incidence. An increase in the number of daily testing was significantly associated with higher COVID-19 incidences (effect size ranged from 2.17–9.96). No factors were significantly associated with daily death rates. Except for the province of Balochistan, a lower daily temperature was associated with a significantly higher daily recovery rate.

**Discussion:**

Environmental factors such as temperature, humidity, wind, and daily sun exposure were not consistently associated with COVID-19 incidence, death rates, or recovery. More policing about precautionary measures and ensuring diagnostic testing and accuracy are needed.

## 1. Introduction

COVID-19 is a novel corona virus which is currently treated as severe pneumonia [1] and reportedly has a very high human-to-human transmission risk [2],[3]. COVID-19 was declared a Public Health Emergency of International Concern (PHEIC) in January 2020 [4] and in March 2020, the World Health Organization (WHO) declared COVID-19 a pandemic and since it has affected 213 countries and two international conveyances [5]–[7]. In Pakistan, the Ministry of Health and government of Pakistan confirmed the first case of COVID-19 on February 26, 2020 in Karachi, the capital of the province of Sindh [8]. The early cases of COVID-19 were reported in pilgrims returning from Iran, Iraq, and Syria who were crossing the bordering city Taftan, Balochistan, and reportedly then travelling to other cities, mainly to Karachi and Gilgit-Baltistan. Within a few weeks time, the numbers of COVID-19 cases soared rapidly; affecting every province and territory in Pakistan. Although initially COVID-19 was mainly reported in travellers, with time there was an increase in community transmission. To deal with the pandemic, and following the international protocol [9], the federal government-imposed lockdowns, travel restrictions, and a ban on mass public gatherings to control the spread of COVID-19.

In Pakistan, a common public believe is that the transmission of COVID-19 will decrease with the rise in temperature during the summer season [10]. Human-to-human contact is key in the transmission of viral illnesses such as influenza, however environmental factors such as humidity, temperature [11],[12], wind [13], and sun exposure [14] play an important role in disease transmission. In other corona viruses such as Middle East Respiratory Syndrome (MERS) [15]–[17] and severe acute respiratory syndrome (SARS) [18],[19], climatic factors are known to affect viral transmission. Climatic factors such as temperature [20] and humidity [21] affects factors such as viral viability and persistence [12], as well as people's behavior and decisions—such as meeting for social gatherings [22]. As compared to the better-known corona viruses, COVID-19 is a novel infection and an exact comparison to transmission and mortality is not available. Comparisons are difficult, potentially due to the differences in transmission such as the rapid spread that created limitations in hospital resources that stretched hospitals beyond their capabilities [23]. Although many symptoms of COVID-19 are the same as the seasonal flu, COVID-19 is different than the common flu in terms of the incubation period, transmission, effects on age groups, and risk factors associated with COVID-19 [24].

With the continuous increase in the number of COVID-19 cases and speculations about the second wave throughout the world, there is need for a high level of action, proper, and thoughtful planning, and management [25]. It will be important to explore the transmissibility of COVID-19 and any association with climatic factors that affect daily incidence, mortality, and recovery rate [26]. As Pakistan does not have uniform weather throughout all provinces and territories, having an understanding about the transmission of COVID-19 during natural environment and climatic changes will be helpful to support decision-making related to disease control. The goal of this article was to explore whether climatic factors such as humidity, wind, temperature, and sun exposure influence the transmission of COVID-19.

## 2. Methods

To conduct this analysis, data was collected from various websites and online sources as reported in previous studies [15],[16],[25],[27],[28], thus ethical approval was not needed. No web scrapping software or programs were used, and data was not altered in any regards. Data was compared to multiple other sources to confirm authenticity.

*Population data:* COVID-19 cases, deaths, recovery and daily testing for each province and, Islamabad, as well as Gilgit-Baltistan and Azad Kashmir were collected online [29]–[31] from March 10 to July 10^th^, 2020.

*Weather data:* Weather data [31]–[34] consisted of daily mean humidity and wind, daily maximum and minimum temperature and sun status. Reports of “broken clouds” and “partly sunny” were merged with “cloudy days”, and “scattered clouds” were merged with “sunny days”.

*Statistical analysis:* Descriptively we reported the cumulative incidence data, deaths, and daily recovery for each province and territory. Continuous variable such as wind (KM/H), humidity (%) and temperature (°C) were reported with mean and standard deviation (SD); whereas categorical data such as daily tests, sunny days, lock down status were reported with numbers and proportions.

Data was analyzed with a log link of negative binomial distribution using the generalized linear mixed model [35],[36]. We chose to use a negative binominal regression model as it is more suitable for count data [36]. The model of fit was determined with the likelihood ratio [37] and the outcome for each territory was reported separately. Besides weather data, the analysis was also adjusted for other variables such as lock down status (lock down vs. smart lock down vs. no lock down) and maximum daily testing status (≥10,000 vs. <10,000). In March-May of 2020 there were few nationwide events that required mass gatherings during which standard operating procedures (SOP) were potentially compromised. The nationwide events included religious events that required mass gatherings, Ramadan (iftar parties), and Eid days. As the incubation period for COVID-19 is 3 to 14 days, we included the following 14 days as a continuation for each nationwide event. First, we analyzed the association of climatic factors, lock down status, maximum daily testing with outcomes variable in univarable analysis. All variables were included in the final analysis for COVID-19 incidence, but the analyses for daily deaths and recovery were not adjusted for lock down status, number of daily testing, and nationwide social events. As humidity correlates with temperature, the interaction between humidity and temperature [11] was included in our secondary analysis. The effect estimates were reported with odds ratio (OR) and 95% confidence intervals (CIs) and statistical analyses were conducted using *SPSS v.25*. statistical software.

## 3. Results

**Figure 1.**
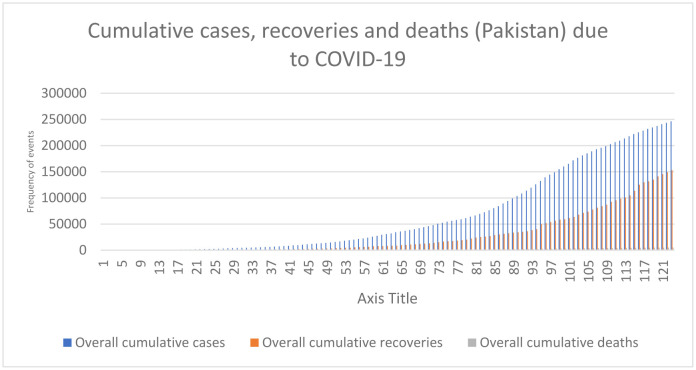
Cumulative cases, deaths, and recoveries due to COVID-19.

**Figure 2.**
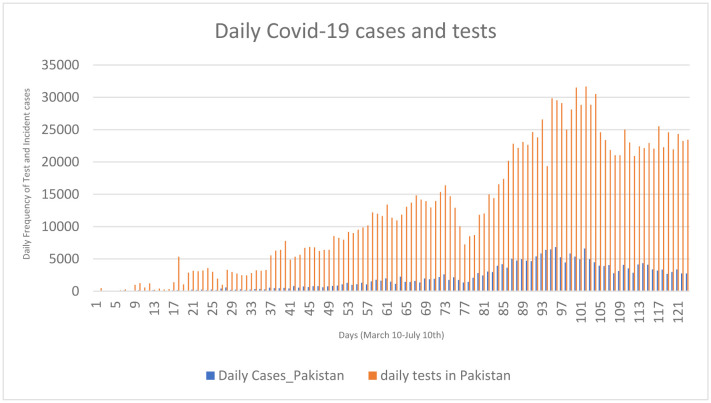
Daily COVID-19 cases and tests. Day 23 = Lock down started in Pakistan; Day 24 = Nationwide event; Day 56 = 10000 tests/day target achieved; Day 61 = Loosening of lock down; Ramadan + Eid = Day 45–76; Day 76 = Eid Day; Day 98 = Beginning of selective lock down.

A summary of cumulative COVID-19 cases, deaths, and recoveries are reported in Table 1. As of July 10^th^, 2020, there were 246,351 confirmed cases of COVID-19—5,123 (2.07%) patients died and 153,134 (62.16%) recovered. Sindh was the most affected province in the country, with a confirmed 102,368 (41.55%) cases, whereas Azad Kashmir region (AZK) had the lowest cumulative cases of COVID-19 (0.62%). Daily mortality rate was highest in Khyber Pakhtunkhwa (KPK) (3.60%) followed by AZK (2.74%). Most recoveries occurred in Gilgit Baltistan (79.93%) followed by Islamabad (74.00%). Figure 1 shows cumulative COVID-19 cases, death, and recoveries. Figure 2 shows the reported daily cases and tests conducted in Pakistan as well as the number of daily COVID-19 cases corresponding with the number of daily tests and trends in daily cases over 123 days. The peaks in daily cases corresponded with the 3–14 day incubation period and were associated with nationwide events and the loosening of lock down restrictions.

**Table 1.** Cumulative incidence, deaths, recovery and mean wind, humidity, temperature minimum (min) and maximum (max).

REGION	Cumulative incidence	Cumulative Deaths	Cumulative Recovery	Total tests	Mean Wind (KM/H)	Mean Humidity (%)	Mean Temperature-Max (C) mean (SD)	Mean temperature-Min (C) mean (SD)	Sunny Day
Sindh	102,368 (41.55%)	1,713 (33.43%) (1.67%) *	59,185 (38.64%) (57.81%) *	254,559 (33.36%)	16.39 (5.37)	61.91 (11.63)	35.23 (3.60)	26.32 (4.25)	98 (79.7%)
Punjab	85,991 (34.90%)	1,985 (38.74%) (2.30%) *	53,836 (35.15%) (62.60%) *	302,720 (39.67%)	11.95 (7.35)	53.41 (15.67)	34.13 (6.19)	22.06 (5.7)	103 (83.7%)
KPK	29,775 (12.08%)	1,074 (20.96%) (3.60%) *	20,271 (13.23%) (68.08%) *	84,842 (11.11%)	14.19 (6.83)	52.24 (15.62)	31.86 (6.59)	20.63 (5.73)	83 (67.5%9)
Balochistan	11,128 (4.51%)	126 (2.45%) (1.132%) *	7,224 (4.71%) (64.91%) *	33,283 (4.36%)	18.24 (11.99)	32.08 (18.07)	28.37 (7.50)	16.86 (7.33)	102 (82.9%)
Islamabad	13,927 (5.65%)	147 (2.86%) (1.05%) *	10,441 (6.81) (74.96%) *	65,634 (8.601%)	12.53 (8.09)	50.83 (20.38)	31.43 (6.20)	20.21 (5.49)	63 (51.2%)
Gilgit Baltistan	1,630 (0.66%)	36 (0.7%) (2.20%) *	1,303 (0.85%) (79.93%) *	12,579 (1.64%)	2.60 (2.49)	58.19 (19.64)	25.60 (6.79)	13.50 (5.95)	60 (48.8%)
Azad Kashmir	1,532 (0.62%)	42 (0.81%) (2.74%) *	94 (0.58%) (58.35%) *	9,437 (1.23%)	4.39 (5.88)	57.35 (18.51)	28.11 (6.21)	16.14 (5.09)	60 (48.8%)
Total	246,351 1109 cases/million	5,123 (2.07%) 23 deaths/million	153,134 (62.16%) 689 recoveries/million	763,054	

Note: KPK: Khyber Pakhtunkhwa; * = proportions from the total cases of each region

**Table 2.** COVID-19 daily cases—univariable analysis [OR (95%CI)].

	SINDH	PUNJAB	KPK	BALOCHISTAN	ISBD	GILGIT	AZK
Wind	1.11 (1.06, 1.15)	1.04 (1.00, 1.07)	1.04 (1.01, 1.07)	1.01 (0.99, 1.03)	1.01 (0.99, 1.04)	1.01 (0.93, 1.10)	0.99 (0.95, 1.02)
Humidity	1.03 (1.01, 1.04)	0.98 (0.97, 0.99)	0.96 (0.95, 0.97)	0.98 (0.97, 0.99)	0.98 (0.97, 0.99)	0.99 (0.98, 1.00)	0.97 (0.96, 0.98)
Temperature Daily Max	1.59 (1.45, 1.74)	1.20 (1.15, 1.24)	1.18 (1.14, 1.21)	1.12 (1.09, 1.15)	1.22 (1.18, 1.26)	1.05 (1.02, 1.08)	1.19 (1.14, 1.23)
Temperature Daily min	1.30 (1.26, 1.34)	1.15 (1.12, 1.18)	1.23 (1.19, 1.28)	1.11 (1.08, 1.15)	1.28 (1.24, 1.33)	1.04 (1.01, 1.07)	1.18 (1.14, 1.23)
Sunny Day (Sunny day; Ref = No)	0.76 (0.49, 1.18)	1.15 (0.71, 1.85)	0.54 (0.37, 0.79)	1.18 (0.74, 1.90)	0.41 (0.29, 0.59)	0.68 (0.47, 0.99)	0.40 (0.28, 0.588)
Lock down stat (Ref = Lock down period)	3.2 (2.12, 4.81)	2.92 (1.94, 4.40)	2.20 (1.46, 3.31)	2.42 (1.60, 3.65)	9.29, (6.13, 14.10)	2.42 (1.57, 3.73)	5.06 (3.15, 8.14)
Smart lock down stat (Ref = Lock down period)	7.87 (4.77, 12.97)	4.76 (2.89, 7.85)	4.22 (2.55, 6.95)	2.51 (1.51, 4.14)	14.11 (8.50, 23.41)	3.09 (1.83, 5.20)	17.71 (10.19, 30.80)
Nationwide Events*	0.74 (0.52, 1.06)	1.12 (0.78, 1.61)	1.00 (0.70, 1.44)	1.72 (1.20, 2.47)	1.53 (1.06, 2.19)	1.08 (0.74, 1.57)	0.80 (0.55, 1.16)
10000/Daily Test	10.28 (7.20, 14.68)	8.43 (5.9, 12.04)	6.88 (4.81, 9.84)	6.58 (4.59, 9.44)	26.46 (18.30, 38.25)	2.81 (1.93, 4.08)	16.64 (10.85, 25.53)
COVID-19 Daily Cases—Multivariable Analysis [OR (95%CI)]							
	SINDH	PUNJAB	KPK	BALOCHISTAN	ISBD	GILGIT	AZK
Wind	1.010 (0.969, 1.053)	1.009 (0.980, 1.039)	0.998 (0.968, 1.028)	1.020 (0.999, 1.041)	0.997 (0.973, 1.022)	0.985 (0.904, 1.073)	0.974 (0.939, 1.010)
Humidity	0.996 (0.977, 1.015)	1.001 (0.983, 1.019)	1.010 (0.992, 1.028)	1.004 (0.989, 1.019)	1.015 (1.004, 1.026)	1.001 (0.991, 1.011)	0.999 (0.984, 1.014)
Temperature Daily Max	1.075 (0.965, 1.197)	1.044 (0.986, 1.106)	1.009 (0.916, 1.106)	1.017 (0.949, 1.090)	1.068 (1.004, 1.136)	1.027 (0.969, 1.089)	1.011 (0.941, 1.085)
Temperature Daily min	1.130 (1.040, 1.228)	1.043 (0.989, 1.101)	1.150 (1.040, 1.271)	1.055 (0.996, 1.117)	1.105 (1.032, 1.182)	0.984 (0.925, 1.046)	1.012 (0.942, 1.087)
Sunny Day (Sunny day; Ref = No)	0.959 (0.587, 1.567)	1.209 (0.720, 2.029)	0.979 0.606, 1.58	0.780 (0.470, 1.296)	1.047 (0.680, 1.612)	1.154 (0.701, 1.900)	0.976 (0.594, 1.604)
Lock down stat (Ref = Lock down period)	1.025 (0.426, 2.465)	0.611 (0.263, 1.421)	0.401 0.169, 0.951	0.344 (0.198, 0.598)	0.674 (0.352, 1.293)	1.550 (0.882, 2.725)	0.636 (0.273, 1.485)
Smart lock down stat (Ref = Lock down period)	2.868 (0.624, 13.175)	1.222 (0.240, 6.217)	0.552 0.111, 2.751	0.180 (0.073, 0.446)	0.754 (0.292, 1.949)	1.608 (0.671, 3.851)	3.243 (1.069, 9.843)
Nationwide Events*	1.963 (0.844, 4.565)	2.262 (0.991, 5.161)	1.710 0.759, 3.855	1.010 (0.589, 1.733)	1.761 (1.008, 3.079)	1.025 (0.624, 1.684)	2.803 (1.581, 4.969)
10000/Daily Test	3.273 (1.115, 9.609)	5.548 (1.955, 15.74)	4.9305 (1.622, 14.833)	9.963 (4.049, 24.514)	9.496 (3.644, 24.748)	2.173 (1.031, 4.580)	9.470 (3.473, 25.819)

Note: * Includes both religious or nation-wide social event that required mass gatherings or when the SOP were potentially not strictly followed. KPK: Khyber Pakhtunkhwa; ISBD: Islamabad; GILGIT: Gilgit Baltistan; AZK: Azad Kashmir. The p-value for the model fit for each region (analysis) was <0.001.

**Table 3.** COVID-19 daily deaths—univariable analysis [OR (95%CI)].

	SINDH	PUNJAB	KPK	BALOCHISTAN	ISBD	GILGIT	AZK
Wind	1.10 (1.05, 1.14)	1.05 (1.02, 1.09)	1.02 (1.00, 1.05)	1.029 (1.00, 1.05)	0.99 (0.96, 1.02)	0.99 (0.84, 1.16)	0.99 (0.92, 1.05)
Humidity	1.04 (1.02, 1.06)	0.99 (0.97, 1.00)	0.96 (0.95, 0.97)	0.96 (0.95, 0.98)	0.98 (0.97, 1.00)	0.98 (0.96, 1.00)	0.97 (0.95, 0.99)
Temperature Daily Max	1.45 (1.29, 1.62)	1.20 (1.15, 1.26)	1.15 (1.11, 1.19)	1.09 (1.05, 1.14)	1.18 (1.12, 1.25)	1.15 (1.07, 1.24)	1.24 (1.12, 1.37)
Temperature Daily min	1.46 (1.36, 1.56)	1.26 (1.20, 1.32)	1.17 (1.12, 1.21)	1.07 (1.03, 1.12)	1.27 (1.18, 1.36)	1.15 (1.07, 1.24)	1.23 (1.12, 1.35)
Sunny Day	0.98 (0.56, 1.41)	1.11 (0.67, 1.81)	0.52 (0.35, 0.78)	1.94 (1.05, 3.56)	0.42 (0.25, 0.69)	0.23 (0.09, 0.56)	0.26 (0.11, 0.58)
COVID-19 Daily Deaths—Multivariable Analysis [OR (95%CI)]							
	SINDH	PUNJAB	KPK	BALOCHISTAN	ISBD	GILGIT	AZK
Wind	1.018 (0.976, 1.062)	1.019 (0.985, 1.054)	1.009 (0.979, 1.040)	1.020 (0.996, 1.045)	0.969 (0.932, 1.007)	0.913 (0.741, 1.124)	0.947 (0.871, 1.029)
Humidity	1.001 (0.980, 1.023)	1.005 (0.985, 1.029)	1.002 (0.982, 1.023)	0.981 (0.961, 1.002)	1.017 (0.994, 1.040)	0.998 (0.970, 1.028)	0.986 (0.955, 1.017)
Temperature Daily Max	1.057 (0.957, 1.168)	1.076 (0.982, 1.16)	1.100 0.997, 1.213	1.085 (0.988, 1.191)	1.100 (1.001, 1.209)	1.026 (0.900, 1.169)	1.108 (0.953, 1.288)
Temperature Daily min	1.421 (1.307, 1.544)	1.194 (1.11, 1.296)	1.065 (0.973, 1.166)	1.002 (0.921, 1.091)	1.197 (1.087, 1.317)	1.105 (0.981, 1.245)	1.143 (1.004, 1.301)
Sunny Day	1.093 (0.640, 1.864)	1.63 (0.94, 2.81)	0.955 0.589, 1.54	2.066 (1.094, 3.902)	0.870 (0.459, 1.650)	0.386 (0.120, 1.240)	0.883 (0.307, 2.536)

Note: The p-value for the model fit for each region was <0.001.

**Table 4.** COVID-19 daily recoveries (negative binomial)—univariable analysis [OR (95%CI)].

	SINDH	PUNJAB	KPK	BALOCHISTAN	ISBD	GILGIT	AZK
Wind	1.15 (1.10, 1.20)	1.01 (0.98, 1.03)	1.02 (0.99, 1.05)	1.01 (0.99, 1.03)	0.97 (0.95, 0.99)	1.03 (0.95, 1.12)	0.93 (0.89, 0.97)
Humidity	1.06 (1.05, 1.08)	0.99 (0.97, 1.00)	0.97 (0.96, 0.98)	0.97 (0.98, 0.98)	0.97 (0.95, 0.98)	0.98 (0.97, 0.99)	0.99 (0.97, 1.00)
Temperature Daily Max	1.92 (1.70, 2.18)	1.42 (1.36, 1.48)	1.29 (1.25, 1.34)	1.26 (1.22, 1.30)	1.43 (1.37, 1.49)	1.06 (1.04, 1.09)	1.13 (1.10, 1.17)
Temperature Daily min	1.76 (1.66, 1.86)	1.19 (1.16, 1.21)	1.34 (1.29, 1.39)	1.20 (1.17, 1.24)	1.58 (1.50, 1.67)	1.10 (1.07, 1.14)	1.31 (1.24, 1.39)
Sunny Day	0.76 (0.49, 1.18)	0.42 (0.26, 0.69)	0.77 (0.52, 1.12)	1.33 (0.83, 2.14)	0.31 (0.21, 0.44)	0.46 (0.32, 0.68)	0.72 (0.49, 1.05)
COVID-19 Daily Recoveries—Multivariable Analysis [OR (95%CI)]							
	SINDH	PUNJAB	KPK	BALOCHISTAN	ISBD	GILGIT	AZK
Wind	1.031 (0.995, 1.069)	0.977 (0.949, 1.006)	0.992 (0.962, 1.023)	1.021 (1.000, 1.043)	0.906 (0.878, 0.934)	1.039 (0.948, 1.138)	0.992 (0.975, 1.009)
Humidity	1.028 (1.012, 1.044)	1.023 (1.003, 1.043)	1.016 (0.994, 1.038)	0.992 (0.978, 1.008)	1.048 (1.027, 1.069)	0.987 (0.975, 0.998)	0.859 (0.809, 0.913)
Temperature Daily Max	1.069 (0.956, 1.196)	1.361 (1.276, 1.445)	1.15 (1.041, 1.269)	1.212 (1.128, 1.303)	1.350 (1.255, 1.452)	0.996 (0.954, 1.039)	1.075 (1.010, 1.144)
Temperature Daily min	1.673, (1.559, 1.796)	1.132 (1.093, 1.172)	1.206 (1.108, 1.314)	1.035 (0.972, 1.102)	1.338 (1.246, 1.436)	1.117 (1.060, 1.177)	1.308 (1.217, 1.405)
Sunny Day	0.989 (0.596, 1.642)	1.858 (0.932, 3.705)	1.51 0.741, 1.789	0.911 (0.531, 1.561)	1.288 (0.819, 2.025)	0.763 (0.502, 1.160)	3.010 (1.751, 5.175)

Note: The p-value for the model fit for each region was <0.001.

The association of various factors with daily COVID-19 cases, deaths, and recovery are reported in Tables 2, 3, and 4. In univariable analysis, humidity was associated with a lower incidence of COVID-19; whereas temperature, lock down status, and number of daily tests were associated with higher daily incidence rate. Humidity was also associated with a decrease in death and recovery rates, whereas temperature was associated with higher death and recovery rates.

*Results of adjusted analysis:* Results of the adjusted analysis for daily new cases, deaths, and recoveries are reported in Table 2, 3 and 4, respectively. In multivariable analysis, except >10,000 tests per day, no other variable was consistently associated with an increase in the number of daily cases (Table 2). With more than 10,000 tests per day, the effect estimates (OR) ranged from 2.173 to 9.963. In multivariable analysis, none of the climatic factors were associated with daily COVID-19 incidence. Daily temperature showed a non-significant trend towards higher daily incidence which became more obvious with reports of daily low temperatures, although low temperatures showed a trend towards higher COVID-19 incidence. Similarly, nationwide events showed non-significant but consistent trends towards increased incidence of infection. Lock down status was not associated with daily incidence rate but mostly showed trend towards high daily incidence rate.

In multivariable analysis, no variable was consistently associated with daily death rates or daily recoveries through all regions (Table 3 and 4). Daily low temperatures showed a trend towards higher recovery rate. The analysis of interaction between temperature and humidity was not significant for all three outcomes.

## 4. Discussion

Our goal was to explore the association of four important environmental factors; wind, humidity, temperature, and daily sun exposure with respect to the transmission of COVID-19. We adjusted our analysis for other important variables such as lock down status, nation wide social events, and daily testing for the COVID-19. The multivariable analysis showed trends for higher odds and increased COVID-19 incidence with low daily temperature. However, high temperature was not significantly associated with the daily COVID-19 incidence. The multivariable analysis showed that the number of daily tests performed was the only factor significantly associated with increased daily incidence of COVID-19. Contrary to common belief, climatic factors—particularly temperature and humidity, did not have consistent association with daily COVID-19 cases. Nationwide social events and lock down status mostly showed a non-significant trend towards increased COVID-19 daily incidences. In the multivariable analysis, no factor showed a consistent association with daily deaths or recoveries throughout all regions. For daily recoveries, daily low temperatures showed a trend towards higher recovery rate.

## 5. Conclusion

To the best of our knowledge, this is the first study to investigate the association between environmental factors, daily tests, nation wide events and lock down status with daily COVID-19 incidence, recoveries, and death dues to COVID-19 in Pakistan. Our study did not show an association between the environmental factors and daily COVID-19 cases, deaths, and recoveries and thus the speculations that the COVID-19 will decrease with warm temperature was not supported. A recent report suggested that weather may alter COVID-19 transmission but may not completely eliminate it [43],[54]. Due to social beliefs among the Pakistani nation, national and local governments need to take rigorous measures to educate people about the importance of social distancing as it remains our strongest method of prevention [42]. Stronger strategies and resources at the national and local levels of government are needed to increase daily testing for COVID-19.More research is needed to explore the true effect of environmental factors such as wind, temperature, humidity, and sun exposure while adjusting for other important factors such as age, gender, socioeconomic factors, underlying comorbidities with daily risks of COVID-19 spread, mortality and recovery, and therefore having more testing, and thus more data, is critical to understanding the true effects and patterns of the virus.

## Strengths and limitations

The key strengths of this study were firstly, the analysis was adjusted for other key variables such as lock down status, number of daily testing, and nationwide mass social gatherings. Secondly, we explored the effect of various climatic factors on different outcomes, which makes the interpretation broad. Thirdly, data was collected on daily basis rather than on a weekly or monthly basis. Limitations of our study are due to the nature of the data utilized, we could not adjust for socioeconomic status, gender, air pollutants and comorbidities. As well, data was collected mainly from publicly available websites compared to data used in a population-based study design, however the data was collected through authentic websites and was compared with multiple sources for accuracy.

## Relevance to literature and current trends of COVID-19

In Pakistan, temperatures did not show significant association with the COVID-19 daily incidence. However, a trend for increased incidence of COVID-19 was noted with lower temperatures. Similarly, inconsistent association of temperature with the COVID-19 incidence was also reported [38] in Hubei, China, where temperature showed a significant reduction in COVID-19 cases, but similar associations with temperature were not reported in other parts of China.39 Our results differed from previous studies [31],[39]–[41] that showed significant association of environmental factors such as temperature, wind, humidity with incidences of COVID-19 deaths and recoveries. One potential explanation for the variation between our results and the previous studies could be due to differences in the analysis plan. Previous studies [31],[39]–[41] analyzed the association between environmental factors and COVID-19 incidence with correlation analysis. We measured association between environmental factors and COVID-19 incidence, death rates and recoveries using regression. Regression is a more suitable analysis method than correlation when predictive association of a variable is desired with the outcome and allows adjusting for other variables that can potentially affect the outcome [42]. Zhu [43] analyzed data using generalized additive method (GAM) and piecewise linear regression to determine the association between COVID-19 incidence and environmental factors. Zhu [43] did not report significant association of temperature with COVID-19 incidence, which concur with our results. Our findings also support the current international trend of COVID-19. On June 8th, 2020 New Zealand declared the nation free from COVID-19 and lifted all restrictions [44]. June is winter in New Zealand with an average temperature of 11.5°C, ranging from 15°C during the day to 8°C at night [45]. If the temperature had influenced COVID-19 spread, then it should not have experienced dramatic reductions in reported COVID-19 cases. New Zealand initially imposed very strict lock down measures and surveillance for COVID-19 that were considered effective in reducing the transmission of COVID-19, but as New Zealand loosened the restrictions new cases of COVID-19 began to emerge [46],[47]. Also, in countries such as India and Brazil with more moderate-to-warm climate, high temperatures did not show effects on reported daily COVID-19 cases [48],[49]. In a lab study by Chin et al [50], the COVID-19 virus became inactive after 30 minutes at 56°C and 5 minutes at 70°C. If temperature has association with COVID-19 transmission, such high environmental temperatures are not likely in day-to-day life. The only finding that consistently showed significant association with daily COVID-19 incidence was increased daily testing. This also aligns with international trends found in countries with higher reported COVID-19 cases like the United States of America, where they have had the resources to perform more testing.

## Clinical implications

In multivariable analyses, only daily testing was associated with higher odds of daily COVID-19 incidence. In most regions, lock down status showed trend for lower odds of COVID-19 incidence and although non-significant, nationwide mass gatherings did show consistent trends towards high incidence. As compared to New Zealand and China, lock down status in Pakistan was not significantly associated with COVID-19 incidence. One possible explanation for the lack of association with lock down status could be due to the majority of the Pakistani nation's social beliefs, potentially people did not follow lock down restriction and SOP strictly [51],[52]. This pattern shows that social distancing remains a vital prevention strategy. COVID-19 is the third major corona virus outbreak after SARS and MERS, both which showed some variation with weather, but it was precautionary measures such as the policies adopted at the international, national, and public level that proved effective in controlling the major outbreaks of these viruses [53].

## References

[1] Zhu N, Zhang D, Wang W (2020). A Novel Coronavirus from Patients with Pneumonia in China, 2019. N Engl J Med.

[2] Sahin AREA, Agaoglu PM, Dineri Y (2020). 2019 Novel Coronavirus (COVID-19) Outbreak: A Review of the Current Literature. EJMO.

[3] Wang L, Wang Y, Ye D (2020). Review of the 2019 novel coronavirus (SARS-CoV-2) based on current evidence. Int J Antimicrob Agents.

[4] WHO (2020). COVID-19 Public Health Emergency of International Concern (PHEIC). Global research and innovation forum: towards a research roadmap.

[5] Lai CC, Wang CY, Wang YH (2020). Global epidemiology of coronavirus disease 2019 (COVID-19): disease incidence, daily cumulative index, mortality, and their association with country healthcare resources and economic status. Int J Antimicrob Agents.

[6] Clark A, Jit M, Warren-Gash C (2020). Global, regional, and national estimates of the population at increased risk of severe COVID-19 due to underlying health conditions in 2020: a modelling study. Lancet Global Health.

[7] Bilgin S, Kurtkulagi O, Bakir Kahveci G (2020). Millennium pandemic: A review of coronavirus disease (COVID-19). Exp Biomed Res.

[8] Waris A, Atta UK, Ali M (2020). COVID-19 outbreak: current scenario of Pakistan. New Microbes New Infect.

[9] Bashir MF, Ma BJ, Bilal (2020). Correlation between environmental pollution indicators and COVID-19 pandemic: A brief study in Californian context. Environ Res.

[10] DAWN (2020). PM Imran hopeful Pakistan's ‘hot and dry’ weather will mitigate virus threat Dawn News TV.

[11] Marr LC, Tang JW, Van Mullekom J (2019). Mechanistic insights into the effect of humidity on airborne influenza virus survival, transmission and incidence. J R Soc Interface.

[12] Park JE, Son WS, Ryu Y (2020). Effects of temperature, humidity, and diurnal temperature range on influenza incidence in a temperate region. Influenza Other Respir Viruses.

[13] Ellwanger JH, Chies JAB (2018). Wind: a neglected factor in the spread of infectious diseases. Lancet Planet Health.

[14] Hobday RA, Dancer SJ (2013). Roles of sunlight and natural ventilation for controlling infection: historical and current perspectives. J Hosp Infect.

[15] Altamimi A, Ahmed AE (2020). Climate factors and incidence of Middle East respiratory syndrome coronavirus. J Infect Public Health.

[16] Gardner EG, Kelton D, Poljak Z (2019). A case-crossover analysis of the impact of weather on primary cases of Middle East respiratory syndrome. BMC Infect Dis.

[17] van Doremalen N, Bushmaker T, Munster VJ (2013). Stability of Middle East respiratory syndrome coronavirus (MERS-CoV) under different environmental conditions. Eurosurveillance.

[18] Darnell MER, Subbarao K, Feinstone SM (2004). Inactivation of the coronavirus that induces severe acute respiratory syndrome, SARS-CoV. J Virol Methods.

[19] Cai QC, Lu J, Xu QF (2007). Influence of meteorological factors and air pollution on the outbreak of severe acute respiratory syndrome. Public Health.

[20] Bi P, Wang J, Hiller JE (2007). Weather: driving force behind the transmission of severe acute respiratory syndrome in China?. Intern Med J.

[21] Casanova LM, Jeon S, Rutala WA (2010). Effects of Air Temperature and Relative Humidity on Coronavirus Survival on Surfaces. Appl Environ Microbiol.

[22] Khan MD, Thi Vu HH, Lai QT (2019). Aggravation of Human Diseases and Climate Change Nexus. Int J Environ Res Public Health.

[23] Faust JS, del Rio C (2020). Assessment of Deaths From COVID-19 and From Seasonal Influenza. JAMA Intern Med.

[24] Centers for Disease Control and Prevention (2020). Similarities and Differences between Flu and COVID-19. Centers for Disease Control and Prevention, National Center for Immunization and Respiratory Diseases (NCIRD).

[25] Adekunle IA, Tella SA, Oyesiku KO (2020). Spatio-temporal analysis of meteorological factors in abating the spread of COVID-19 in Africa. Heliyon.

[26] Parodi SM, Liu VX (2020). From Containment to Mitigation of COVID-19 in the US. JAMA.

[27] Ma Y, Zhao Y, Liu J (2020). Effects of temperature variation and humidity on the death of COVID-19 in Wuhan, China. Sci Total Environ.

[28] Luo W, Majumder MS, Liu D (2020). The role of absolute humidity on transmission rates of the COVID-19 outbreak. medRxiv.

[29] Government of Pakistan (2020). Pakistan Cases Details.

[30] Worldometer (2020). Coronavirus Cases: Pakistan.

[31] Rosario DKA, Mutz YS, Bernardes PC (2020). Relationship between COVID-19 and weather: Case study in a tropical country. Int J Hyg Environ Health.

[32] Pakistan Meteorological Department, Government of Pakistan (2020). Pakistan Meteorological Department.

[33] Time and Data (2020). Weather in Pakistan.

[34] Weather (2020). Pakistan Weather.

[35] Yun J, Greiner M, Holler C (2016). Association between the ambient temperature and the occurrence of human Salmonella and Campylobacter infections. Sci Rep.

[36] Hernandez E, Torres R, Joyce AL (2019). Environmental and Sociological Factors Associated with the Incidence of West Nile Virus Cases in the Northern San Joaquin Valley of California, 2011–2015. Vector Borne Zoonotic Dis.

[37] Crainiceanu CM, Ruppert D (2004). Likelihood ratio tests for goodness-of-fit of a nonlinear regression model. J Multivar Anal.

[38] Qi H, Xiao S, Shi R (2020). COVID-19 transmission in Mainland China is associated with temperature and humidity: A time-series analysis. Sci Total Environ.

[39] Sarmadi M, Marufi N, Kazemi Moghaddam V (2020). Association of COVID-19 global distribution and environmental and demographic factors: An updated three-month study. Environ Res.

[40] Şahin M (2020). Impact of weather on COVID-19 pandemic in Turkey. Sci Total Environ.

[41] Iqbal MM, Abid I, Hussain S (2020). The effects of regional climatic condition on the spread of COVID-19 at global scale. Sci Total Environ.

[42] Tanni SE, Patino CM, Ferreira JC (2020). Correlation vs. regression in association studies. Jornal Brasileiro de Pneumologia.

[43] Zhu Y, Xie J (2020). Association between ambient temperature and COVID-19 infection in 122 cities from China. Sci Total Environ.

[44] BBC (2020). New Zealand lifts all Covid restrictions, declaring the nation virus-free.

[45] Weather2Visit Auckland Weather in June, What's the weather like in Auckland, New Zealand in June 2020?.

[46] Lewis D (2020). We felt we had beaten it': New Zealand's race to eliminate the coronavirus again. Nature.

[47] Perry N (2020). After 102 days coronavirus-free, New Zealand reports 4 new cases. Global News.

[48] India TTo (2020). Coronavirus: India crosses 80,000 cases in a day, first country to do so. The Times of India.

[49] BBC News (2020). Coronavirus: India surpasses US for highest single-day rise in Covid-19 cases.

[50] Chin AWH, Chu JTS, Perera MRA (2020). Stability of SARS-CoV-2 in different environmental conditions. Lancet Microbe.

[51] The New York Times (2020). Imams Overrule Pakistan's Coronavirus Lockdown as Ramadan Nears.

[52] Farmer B (2020). Pakistan will reimpose lockdown if residents don't observe safety rules, ministers warn. The National AE.

[53] Whiting K (2020). Two experts explain what other viruses can teach us about COVID-19—and what they can't. The World Economic Forum COVID Action Platform.

[54] Jonathan Lambert (2020). Warm weather probably won't slow COVID-19 transmission much. Any seasonal benefit can be canceled by humanity's vulnerability to the virus, a study suggests. Science News.

